# Towards implementing coordinated healthy lifestyle promotion in primary care: a mixed method study

**DOI:** 10.5334/ijic.1741

**Published:** 2015-08-18

**Authors:** Kristin Thomas, Preben Bendtsen, Barbro Krevers

**Affiliations:** Department of Medical and Health Sciences, Linköping University, Linköping, Sweden; Department of Medical Specialist and Department of Medical and Health Sciences, Linköping University, Motala, Sweden; Department of Medical and Health Sciences, Linköping University, Linköping, Sweden

**Keywords:** healthy lifestyle promotion, primary care, process evaluation, implementation, General Theory of Implementation, mixed methods

## Abstract

**Background:**

Primary care is increasingly being encouraged to integrate healthy lifestyle promotion in routine care. However, implementation has been suboptimal. Coordinated care could facilitate lifestyle promotion practice but more empirical knowledge is needed about the implementation process of coordinated care initiatives. This study aimed to evaluate the implementation of a coordinated healthy lifestyle promotion initiative in a primary care setting.

**Methods:**

A mixed method, convergent, parallel design was used. Three primary care centres took part in a two-year research project. Data collection methods included individual interviews, document data and questionnaires. The General Theory of Implementation was used as a framework in the analysis to integrate the data sources.

**Results:**

Multi-disciplinary teams were implemented in the centres although the role of the teams as a resource for coordinated lifestyle promotion was not fully embedded at the centres. Embedding of the teams was challenged by differences among the staff, patients and team members on resources, commitment, social norms and roles.

**Conclusions:**

The study highlights the importance of identifying and engaging key stakeholders early in an implementation process. The findings showed how the development phase influenced the implementation and embedding processes, which add aspects to the General Theory of Implementation.

## Background

Lifestyle-related illness and disease, such as cardiovascular diseases, cancers and diabetes, are a leading cause of death worldwide. Health care organizations are therefore encouraged to integrate healthy lifestyle promotion in routine care [1–4]. Healthy lifestyle promotion is the promotion of an active lifestyle, healthy eating habits, tobacco cessation and moderate drinking of alcohol including screening for risky lifestyle, brief advice about healthy living and extended counselling. Primary care has been proposed as a suitable setting due to its capacity to reach a large number of patients, its credibility and it is the first point of contact for many patients [5, 6]. However, the provision of lifestyle promotion in primary care has been suboptimal [7]. A study investigating lifestyle promotion practice in primary care between 1975 and 2008 showed that only 6–13% of consultations included lifestyle advice [8]. Barriers for healthy lifestyle promotion practice include beliefs, confidence, heavy workload and attitudes among staff as well as limited resources, restricted reimbursements and lack of referral resources [5, 9–11]. Coordinated care has been found to improve continuity of care, reduce mortality, reduce inappropriate hospital admissions, improve quality of services and reduce costs in mental health and diabetes care [12, 13]. Coordinated care could be one way to facilitate lifestyle promotion practice, and it is repeatedly advocated in policy documents [14].

Coordinated lifestyle promotion in primary care could entail screening, brief advice and referral to in-house- or community-based resources [14, 15]. The aim of coordinated care is to improve access, efficiency and quality of care [16]. Coordinated care has found to be characterized by the participation of the patient and numerous health care staff; interdependence; an understanding of the role of others; and the exchange of information [17, 18]. Typically, interventions that have been used to achieve coordinated care have involved multi-professional teams, care management and disease management. Studies have shown that coordinated care can increase the quality of services and reduce costs in mental health and diabetes care [12, 13]. However, few studies have investigated how to best organize and implement coordinated care, specifically in the area of lifestyle promotion [14, 19–21].

There is an increasing amount of evidence on the challenging nature of implementing organizational interventions, such as coordinated care, in health care organizations [19]. Collaboration between professional groups is one important aspect of coordinated care. Studies have shown that collaboration has been difficult to achieve due to conflicts between professional groups, slow-moving decision making and confusion about roles and responsibilities [22–26]. An added challenge is the complex nature of many health care interventions, which require instrumental as well as conceptual change among individual practitioners, professional groups and potentially patients [24]. Coordinated care is complex and often requires change on several levels: behavioural (e.g. referral to specialized staff or multi-professional teams), cognitive (e.g. attitudes) and relational (e.g. collaboration between professions). Although there is evidence to support coordinated care, how coordinated care is implemented in routine practice under real-world conditions needs more research [16, 27]. There are several theories about important determinants for implementation of which the General Theory of Implementation is one [28]. The General Theory of Implementation operationalizes implementation as a continuous interaction between the qualities of a new practice, the social system in which implementation occurs and the agents (individuals or groups) within that social system. Considering that coordinated care implies a high amount of social interaction, the General Theory of Implementation is a suitable framework for analysis of the implementation process of such care.

### Aim

This study aimed to evaluate the implementation of a coordinated healthy lifestyle promotion initiative in a primary care setting. The General Theory of Implementation was used as a framework in the analysis.

## Methods

### Study design

**Figure 1.**
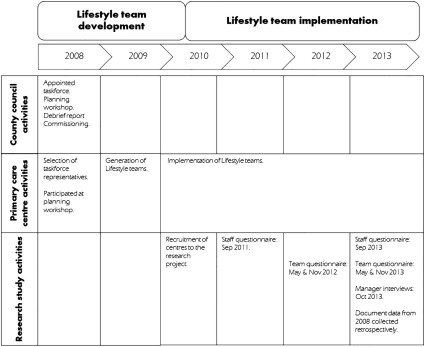
Activities carried out by the actors involved during the period under investigation.

A mixed method, convergent, parallel design was used to gain a comprehensive understanding of the implementation process and outcomes [29]. Three primary care centres took part in the two-year research project. Data collection involved individual interviews and document data on the implementation process and outcomes (retrospective) and two questionnaires investigating team performance and staff readiness to change (prospective). Figure 1 shows the implementation activities carried out by the County Council and primary care centres and the research activities carried out by the research group.

### Coordinated care initiative and setting

The study was performed in Östergötland County in southeast Sweden, with approximately 440,000 inhabitants. The County Council has administrative and economical responsibility for publicly financed health care and is divided into four primary care divisions. These divisions have their own management group and are responsible for the provision of medical care, preventative services and rehabilitation. In 2008, one management group commissioned 10 primary care centres to implement a team-based coordinated care initiative including four components: (1) multi-professional teams, (2) team managers, (3) team meetings at least every 6 weeks and (4) in-house referral procedures for patients with health risk behaviours, i.e. sedentary lifestyle, risky alcohol consumption, poor nutrition or tobacco consumption. This coordinated care initiative is referred to as the lifestyle team.

Coordination of healthy lifestyle promotion involved screening and offering brief advice to at-risk patients in general practice, and if necessary, referring to team members (specialized in healthy lifestyle promotion).

### Participating centres

The highest performing primary care centre in terms of implementing the lifestyle teams was chosen. Degree of implementation was in relation to the four commissioned components (see above) using performance data from the County Council. Three of the 10 commissioned centers were invited to take part in the project. These three centers had started implementing a lifestyle team, which made these centers suitable for inclusion in the study.

A recent study compared these lifestyle team centres with control centres over a period of two years. Centers with lifestyle teams did not show larger proportion of patients receiving healthy lifestyle promotion. Conceptual differences between study groups were found, however, with these differences remaining over time. Lifestyle team centres were more likely to agree that lifestyle promotion issues were prioritized at their workplace and that there were sufficient competency at their workplace regarding lifestyle promotion [30].

The three lifestyle teams which took part in the study varied in size throughout the two-year research period: team A (6–7 members), B (10–15 members) and C (10–11 members). The teams predominantly consisted of women but all teams had 1–2 male members. All teams consisted of behavioural therapists, dieticians, district nurses, specialized nurses and practice managers. Two teams also included physicians (A and B) and medical secretaries (B and C). The number of staff at the centres also varied in size throughout the research period: centre A (28 staff members), B (31–35 staff members) and C (31–35 staff members).

### Data sources and collection

The data sources consisted of the staff questionnaire, the team questionnaire, manager interviews and document data. Figure 1 shows a timeline of the activities under investigation, data collection and data sources.

#### Staff questionnaire

Readiness for change was assessed with the context scale of the Organizational Readiness to Change Assessment [31]. A 4-point Likert response scale from “strongly disagree” to “strongly agree” was used. The context scale contained 23 items with six subscales:

- Staff culture (four items), e.g. staff readiness to change
- Opinion leaders (four items), e.g. encourage and support changes
- Resources (four items), e.g. necessary support, staffing and budget
- Leadership culture (three items), e.g. senior leaders reward innovation and creativity
- Leadership practice (four items), e.g. senior leaders clearly define areas of responsibility
- Evaluation (four items), e.g. senior leaders provide staff with feedback

An e-mail including information about the aim of the study and a link to the questionnaire was sent to all eligible staff. Two reminders were sent via e-mail 2–3 weeks after the initial e-mail. Participants completed and returned the questionnaires electronically. Participants included physicians, nurses, dieticians and behavioural therapists including practice managers and team members. The questionnaire was conducted at two time points: September 2011 and September 2013.

#### Team questionnaire

A short team questionnaire was generated by the researchers based on a thorough review of the research literature. Twelve items were generated from validated instruments and aimed to capture important factors for team performance [32]. The items were categorized by the researchers as follows:

- Structure (four items): composition, goals, roles and values
- Process (four items): conflict management, communication, cohesion and reflection
- Team effectiveness (four items): integration of lifestyle promotion practice at the center, primary and secondary referral practice of the teams, and shared understanding of the teams’ purpose at the center

The layout of the questionnaire was in the shape of a 12-armed star with statements presented at the end of each arm. The actual arms represented visual analogue scales with labelled scores between 0 and 10 to guide completion (0, disagree; 10, agree). The layout was designed to facilitate completion and prevent attrition. The items and layout of the questionnaire were reviewed by an expert panel and pilot tested with other lifestyle teams not involved in this study. Revisions were done to reach face and content validity, e.g. making the wording more context specific by substituting “group” with “lifestyle team”.

All team members were invited to complete the questionnaire. The team questionnaire was conducted at four time points at six-month intervals between May 2012 and November 2013. Information about the aim of the study, questionnaires and self-addressed envelopes were posted to the centres. A contact person at each centre distributed the material to all team members. Participants completed the questionnaires individually and anonymously and returned them by post. Two reminders were sent via e-mail to the contact person 2–3 weeks after the initial invitation.

#### Manager interviews

A semi-structured interview guide, developed by the researchers, contained questions on the implementation process: implementation activities, challenges, successes and outcomes. All managers (practice and team managers) were sent an e-mail invitation accompanied by information about the aims of the study and confidentiality. All managers took part in interviews. The interviews were conducted by telephone, audio-recorded and transcribed; each interview lasted for about 30 minutes. Data were collected prospectively between September 2011 and October 2013.

#### Document data

Document data from the debriefing report and minutes of planning workshops were collected from the County Council. Data from the debriefing report included background information on the lifestyle teams; description of the status of lifestyle promotion practices in primary care in the region; recommendations for improvement and commissioning of the lifestyle teams. Minutes from the workshop contained information about the planning of the lifestyle team practice. The document data were collected retrospectively in 2013.

### Data analyses

Qualitative data analysis was mainly a joint effort between KT and BK. The analyses were an iterative process including initial analyses by KT, followed by discussions between KT and BK, further analyses and discussions. KT did the initial quantitative analysis and preliminary analyses were presented to BK and PB and were discussed recurrently with all authors.

#### Theoretical framework

The General Theory of Implementation [28] was used as a conceptual tool during the analysis of both qualitative and quantitative data to identify, describe and explain salient factors on the implementation process and outcomes. The General Theory of Implementation argues that implementation can be explained by four constructs: potential, capacity, capability and contribution. These constructs refer to the potential among agents (individuals or groups); the capacity of the social system to implement and embed a new practice; the capability of agents to operationalize the new practice; and the contribution, i.e. what agents do to implement and embed the new practice. Each construct consists of core components or dimensions (refer to Table 1 for a full list of dimensions). The General Theory of Implementation is concerned with the implementation (bringing a practice into action); embedding (incorporating practices in everyday work) and integration (sustaining practices) [28]. The General Theory of Implementation was believed to be a suitable theory because it allowed for the investigation of contextual, relational and individual factors on implementation.

#### Quantitative data

Based on the staff questionnaire, differences between centres were analysed for each subscale of the Organizational Readiness to Change Assessment context scale using analysis of variance and mean scores.

Median scores for each factor important to team performance and the time point were calculated for each index of the team questionnaire: process, structure and team effectiveness [32]. The convention in Ware et al. [33] was applied to handle missing values. An individual had to answer at least half of the questions on a scale for their score to be included in the analyses.

#### Qualitative data

Qualitative content analysis was used for analysing the qualitative data [34]. A predefined analysis scheme was used based on the General Theory of Implementation constructs and dimensions. All data were coded according to the constructs, and then according to the dimensions. Data that did not match the scheme, but were still assessed as relevant to the study aim, were coded as “other”. In the further analysis process, these codes formed a new category that was labelled based on its content. Throughout the analyses the aim was to identify data important for the purpose of the study, i.e., aspects that illustrated the implementation process or explained outcomes.

### Ethical statement

The study was conducted with the approval of the local Central Ethical Review Board (DNR: IMH-2009-00335).

## Results

### Participation rates

All five managers took part; at one centre, the practice manager and the team manager were the same person. All were women aged between 55 and 59 years.

Overall, on average 19 (66%) team members responded to the team questionnaire. The response rate varied between 15 (50%) and 22 (79%) team members during the research period. The mean age was 49 years (standard deviation, 10 years).

In 2011, 77 (78%) practitioners responded to the staff questionnaire; the mean age was 48 years (standard deviation, 11 years) and 83% were women. In 2013, 69 (74%) responded; the mean age was 48 years (standard deviation, 12 years) and 85% were women. Physicians, nurses, dieticians and behavioural therapists took part.

### Main findings

The four General Theory of Implementation constructs and corresponding dimensions were used to analyse the implementation processes and outcomes [28]. If not otherwise stated, the main data source in the results was the interview data. The data coded as “other” formed a fifth category, “Development phase”, which is presented below. This is followed by findings for each General Theory of Implementation construct including the corresponding dimensions. Table 1 summarizes the key findings for each category and their data source.

#### Development phase

The development phase included information on the origin, development and objectives of the lifestyle teams. The lifestyle teams were the result of a dialogue between the primary care management group and staff representatives. Although the implementation was commissioned, staff played an active role in the development phase. Current practice routines were analysed by carrying out status evaluations and needs assessments and identifying barriers and facilitators for healthy lifestyle promotion. Barriers included limited knowledge exchange between professions; difficulties in identifying and reaching at-risk patients; and lack of standardized patient pathways for lifestyle promotion. Actions and plans for an improved, coordinated, lifestyle promotion practice were formulated, including staffs’ request for a flexible practice model that could be adapted for each centre. The staff who participated in the development phase were mainly future team members and individuals who worked with lifestyle-related illness and disease.

After a period of development, 10 centres were commissioned to implement lifestyle teams. A lifestyle team protocol stipulated implementation of four components: (1) multi-professional teams, (2) team managers, (3) team meetings at least every 6 weeks and (4) in-house referral procedures for patients with health risk behaviours, i.e. sedentary lifestyle, risky alcohol consumption, poor nutrition or tobacco consumption. The protocol aimed to reduce barriers by coordinating practice, promoting knowledge exchange and making teams and their members an explicit referral resource. The aim was to use existing resources to coordinate healthy lifestyle promotion at the centres. No additional funding was provided and specialized staff was already based at the centres.

#### Capability

**Figure 2.**
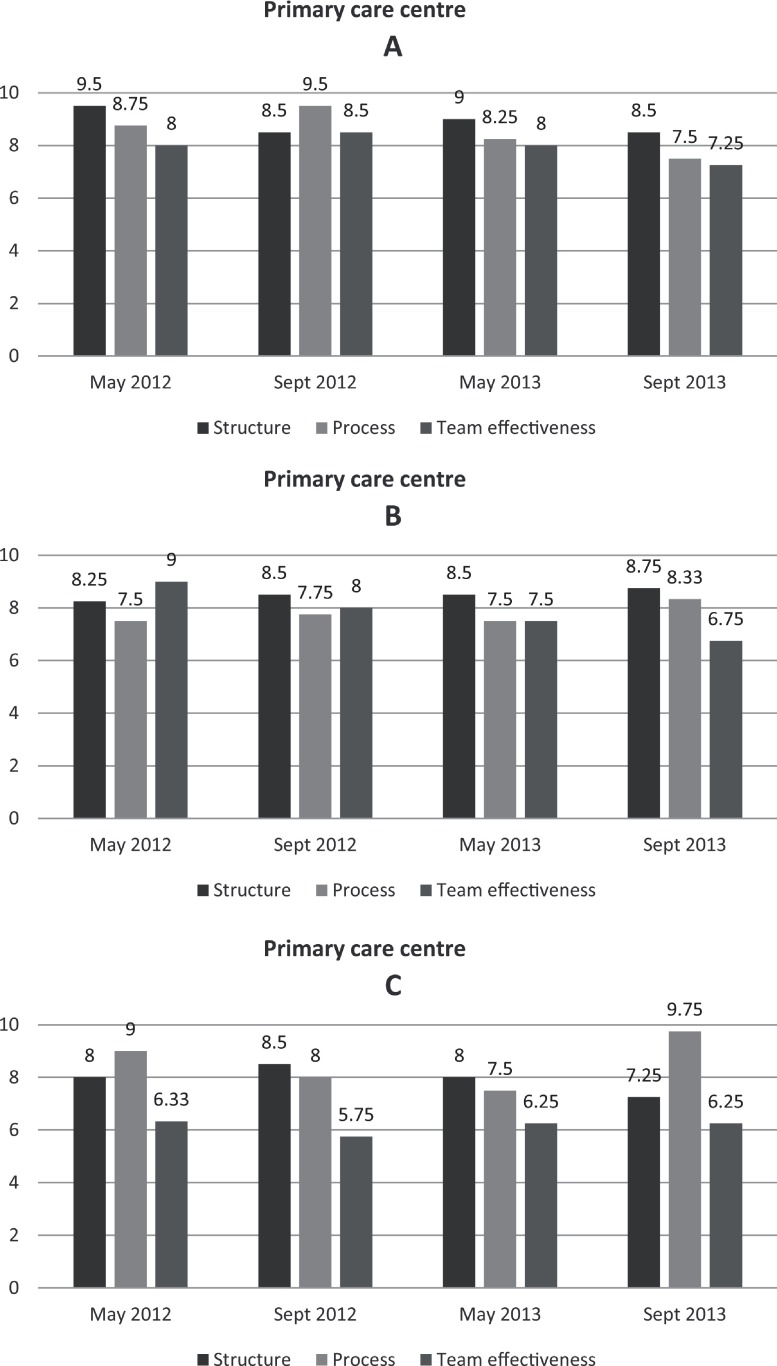
Lifestyle team members perception concerning team performance factors: structure, process and team effectiveness. Median scores for each primary care centre at four time points.

Capability entailed the workability and integration of the lifestyle teams, i.e. how the teams could be operationalized and incorporated at the centres. The protocol had seemingly a high degree of workability. The protocol could be implemented without major changes in practice routines using the existing structures and resources at the centres. However, the protocol simply offered a structure for the lifestyle teams, whereas additional, more complex, coordination activities were required to integrate the teams and their services at the centres. The managers reported that referral procedures were perceived as an implicit understanding among staff and used inconsistently. In the team questionnaire, team members scored consistently high on within-group factors such as team process and team structure but had the lowest scores on without-group items such as team effectiveness at the centres (Figure 2). Thus, the workability and integration of the lifestyle teams in routine practice were challenging and compromised the implementation process.

#### Capacity

Capacity comprised of cognitive and material resources, and social roles and norms that facilitated or hindered implementation. The teams’ cognitive resources had the potential to facilitate implementation. Cognitive resources included relevant competency for lifestyle promotion and could be a resource for staff and a support for patients. Implementation was hindered by limited material resources such as time and staff shortages. The lifestyle teams struggled to prioritize long-term activities such as evaluation and goal setting and staff were reported to struggle to prioritize lifestyle promotion during consultations. Furthermore, social norms and roles among staff and patients hindered implementation. Managers reported that staff perceptions sometimes questioned the work of the lifestyle teams by assigning primary care a role of treating rather than preventing illness and disease. Similarly, managers perceived that most patients seek care due to a specific illness, not for preventative purposes. In contrast, social norms and roles among team members were congruent with the work of the lifestyle team and facilitated its implementation. Thus, the capacity to implement lifestyle teams included both barriers and facilitators.

#### Potential

Potential entailed individual intentions and shared commitment among team members and staff to implement the lifestyle teams and included readiness to change, attitudes, motivation and beliefs. Among team members, implementation was facilitated by their individual intentions and shared commitment. Motivation and positive attitudes towards the concept of lifestyle teams occurred among future team members during the development phase because individuals were part of how the teams were to be developed and implemented. Also, beliefs and attitudes associated with the lifestyle teams were consistent with team members’ existing work on preventative care. According to the staff questionnaire, the centres had a high level of general readiness to change, which suggests shared commitment for implementation. The readiness to change remained over time and there was no difference between centres (Table 2). However, managers reported that it was difficult to consistently engage staff, specifically physicians, in lifestyle promotion and referral procedures which indicate that there was limited shared commitment to implement the lifestyle teams. Thus, there was a possible difference in potential between team members and staff that compromised the implementation process.

#### Contribution

Contribution included the work that the staff, team members and centres did to implement the lifestyle teams in terms of cognitive participation, collective action, coherence and reflective monitoring. Cognitive participation, i.e. how agents got involved and stayed committed to implementation, differed between team members and staff. Team members’ commitment was facilitated by attitudes and social norms that were congruent with the work of the lifestyle team. Also, during the development phase, most team members had contributed to the design of the lifestyle teams and were thus part of the implementation process at an early stage. Commitment among staff was challenged by incongruent social norms and limited time to prioritize lifestyle promotion. Although commitment among staff increased over time, illustrated by attendance and participation at staff meetings on lifestyle promotion, it was still difficult to achieve tangible practice changes such as increased referral rates. However, managers reported that the multi-professional representation in the teams facilitated commitment among staff through information flow and buy-in among professional groups. Managers were key individuals in promoting commitment; this applied especially to practice managers because they had a mandate to put pressure on staff if necessary. Thus, cognitive participation differed between team members and staff due to differences in potential, capacity and their roles during the development phase of the lifestyle teams.

*Collective action*. How skills and resources were mobilized was mainly influenced by the challenges in capability and differences in potential between team members and staff. Team members continuously tried to compensate for these challenges by enacting activities aimed at operationalizing and integrating the lifestyle teams at the centres and increasing commitment among the staff. These activities continuously changed and included monitoring practice rates and giving feedback to staff; increasing awareness and mobilizing staff and patients; networking with external agents; and promoting positive group processes within the lifestyle teams. Thus, collective action required continuous effort from team members and managers; the focus was on managing implementation challenges, capability and differences in potential. However, the protocol offered a structure for the organization of lifestyle promotion at the centres. The referral procedures that were put in place clarified roles and responsibilities by, e.g., a referral coordinator or predefined criteria.

*Coherence*. Making sense of the lifestyle teams was an informal and continuous process among team members. This process mainly entailed balancing external and internal expectations about the purpose and role of the lifestyle teams (staff and patients vs. team members). The aim was to reduce the differences in commitment, resources and social norms. The flexible nature of the protocol allowed for this process. Furthermore, despite implementation challenges, managers perceived the lifestyle teams to be the vehicle for lifestyle promotion practices and team meetings to be an important forum for discussing issues, which drove the implementation forward.

*Reflexive monitoring*. Appraising and evaluating the work was also an informal and continuous process among team members. During this process, the teams redefined their purpose and ambitions. Ambitions changed from aiming to screen every patient for health risk behaviours to screening specific patient groups. As well as focusing predominantly on hypertension, diabetes and lifestyle promotion, the teams added healthy aging, fall prevention and mental health promotion to their work. Redefinition was a response to observations that the teams’ work did not achieve the original ambitions and hopes. It was difficult to systematically evaluate the work due to limited resources (e.g. time). Pay-per-performance feedback from the County Council, e.g. exercise referral, was used to monitor progress at the centres. Thus, during reflexive monitoring and coherence work, team members tried to accommodate the capability of the protocol, the capacity and potential of the centres and future demands.

In summary, structural opportunities for coordinated care were implemented at the centers, e.g. referral procedures. However, conditions for implementation differed between team members and staff making the embedding of the teams (incorporating practices in everyday work) challenging. Conditions for implementation included: resources, individual and shared commitment, social norms and roles and participation in the development of the teams.

## Discussion

We set out to evaluate the implementation of a real-world coordinated care initiative using the General Theory of Implementation [28]. The initiative comprised lifestyle teams that aimed to coordinate lifestyle promotion practice in primary care. The original protocol was implemented at the centres; however, the role of the teams as a referral resource was used inconsistently. Efforts to embed the lifestyle teams were hindered by limited material resources, limited shared commitment, and inconsistent social norms and roles. Findings showed that the development phase influenced the implementation and embedding of the lifestyle teams. The implementation process differed between team members and staff, mainly due to differences in potential and capacity. The General Theory of Implementation was found to be a suitable theory; the findings show that the constructs of capability, capacity and potential together influenced the contribution in relation to the implementation process and the outcomes. However, we cannot say if this was due to the lack of embedding of the teams or not.

### Implementation process

The implementation process of the lifestyle teams differed between staff and team members due to differences in preconditions, e.g. resources, attitudes, motivation and commitment. Also, the participation of the two groups differed during the development phase, which may have influenced their continued commitment during the implementation process. The source of an intervention, whether it is developed externally or internally, and whose problem it solves has been proposed to influence implementation [35]. In our study, the definition of the problem (suboptimal lifestyle promotion practice) and the urgency for change evolved during the development phase, which predominantly involved future team members. Thus, team members could have developed positive preconditions that were difficult to translate to staff during the implementation process when practice change also had to compete with other demands and limited resources. The importance of participation among stakeholders has been highlighted in the literature [36, 37]. For example, a clear shared vision among all stakeholders has been shown to be important to facilitate care coordination [37]. However, our study highlights the importance of also identifying stakeholders outside the immediate coordinated care intervention, i.e. users among staff and patients.

Interestingly, readiness to change among staff remained over time and there was no difference between centres. In contrast, managers reported difficulty in engaging staff, specifically physicians, in lifestyle promotion and referral of patients. These contradictory findings can be explained by the fact that the questionnaire investigated general change rather than the implementation of the lifestyle teams specifically.

Furthermore, the findings show how team members tried to embed the lifestyle teams and their services by adapting ambitions and activities to the conditions at the centres. Adaptations to interventions during the implementation process are common and sometimes even necessary to fully embed new practices [28, 35]. Changes have been found to emerge to make interventions fit a specific context by adjusting to local resources and circumstances [38]. It has been argued that factors such as leadership and norms, values, attitudes and motivation towards the intervention influence adaptations [39]. It is important to investigate why and how changes occur, and their consequences for the implementation outcome, in order to understand the effort required to embed a new intervention within a social system. In our study, the flexibility of the protocol allowed for adaptations. Simultaneously, the predominantly structural nature of the protocol, together with the challenges in engaging the staff, required team members to make adaptations, e.g. to the coordination activities they used and their ambitions.

### Implementation outcomes

The findings show that the four components of the lifestyle team protocol were implemented at the centres. Also, team factors important for team performance, i.e., process and structure, were implemented. Thus, there were opportunities for coordinated care at the centres. However, the findings show that the teams and their services were not fully embedded at the centres. The General Theory of Implementation argues that for an intervention to be embedded, capability, capacity, potential and contribution must be sustained [28]. The findings are in line with the arguments of the General Theory of Implementation; there was limited commitment among the staff and limited resources to prioritize lifestyle promotion among the staff and long-term quality improvement work within the teams. These factors made it challenging to implement and embed the teams at the centres. Critical factors in the implementation process were shared commitment, social norms and roles, attitudes, motivation and resources, with managers having a key role in driving the implementation process forward.

Although structural changes may create potential for coordinated care, fundamental cultural and relational changes may be necessary to fully embed coordinated care [36, 40–42]. Other studies have shown that individual attitudes and beliefs influence lifestyle promotion practice in primary care [10, 41]. Individual differences in lifestyle promotion practice among staff have an impact on coordinated care because limited screening or brief advice can result in limited referral to specialized staff. Another study [40] investigated coordinated care in multiple primary care centres and found that fundamental changes were necessary regarding how professional roles were perceived. It was argued that even a single health care centre consisted of several subgroups and cultures that had to accommodate each other in an implementation process. Leadership was argued to play a critical role in supporting staff to make these changes to culture and identity that are necessary for implementing coordinated care. Our study clearly shows how differences in two subgroups influence the implementation process.

### Implications for practice

This study identified factors that facilitated and hindered coordinated lifestyle promotion. The findings may inform future design and implementation efforts for coordinated lifestyle promotion in primary care. One main factor to take into account is the potential differences between subgroups within an implementation context. Specifically, potential differences regarding the resources that are available; and differences in attitudes, motivation, norms and roles regarding the new practice must be identified. Findings showed how events during the development phase, e.g., level of participation, influenced subsequent conditions for implementation. That is, preconditions, such as positive attitudes towards the lifestyle teams, emerged during the development phase. Disparities between stakeholder groups could be reduced or overcome by taking variations into account when developing a new practice model or by involving all stakeholders if feasible.

### Methodological considerations

The General Theory of Implementation allowed for a comprehensive analysis of implementation [28] and was a useful tool to identify facilitators and hindrances to implementation and investigate the relationship between important factors. However, data coding was on occasion challenging due to potential overlap of the constructs. Also, it was quite evident that some dimensions were similar in this particular dataset. For example, coherence work and reflexive monitoring showed similar features in that they were continuous informal processes aiming to manage the differences between staff and team members.

The lifestyle team initiative was suitable for investigating the implementation of practice change under real-world conditions. The study design, which spanned 2 years, and the 5-year implementation process enable the study to contribute to knowledge of long-term implementation. The use of mixed methods was an appropriate approach to follow an implementation process over such a long period and investigate a broad range of aspects. Unfortunately, no suitable validated instrument to investigate team performance was found. As the instrument was used at four time points, feasibility of administration and acceptability among respondents were essential. The research team carried out an extensive search for suitable instruments without finding a short instrument that would capture the study aim and be feasible for the study design that was used. However, the team questionnaire was carefully developed, based on the literature and was considered to reach face and content validity.

One limitation of the study is that interviews were only conducted with key informants (all managers). Future studies can include staff interviews to gain a more complete picture of the implementation of coordinated care. Data showing the impact of the team initiative are only briefly reported in the methods section as the focus of this study was to evaluate the implementation process. Reported data from a national patient survey may give a glimpse of the impact of the initiative on lifestyle promotion practice. Furthermore, the teams had no routine for registering the number of patients screened for risky lifestyle at the centres or the number of referrals to the teams which made it difficult to evaluate the impact of the lifestyle teams on behavioural and clinical outcomes specifically.

## Conclusions

The study demonstrates that the General Theory of Implementation is a useful tool for analysing the implementation processes. The findings may add to the theory by showing how the development phase influenced the implementation and embedding of the lifestyle teams. The implementation process differed between subgroups at the centres due to differences in preconditions. Structural opportunities for coordinated care were implemented at the centres, e.g. referral procedures. However, efforts to embed the lifestyle teams at the centres were hindered by limited resources, limited shared commitment, and social norms and roles that were inconsistent with the work of the lifestyle teams. The study highlights the importance of identifying and engaging key stakeholders, including patients, early in an implementation process in order to embed a new practice.

## Figures and Tables

**Table 1.**
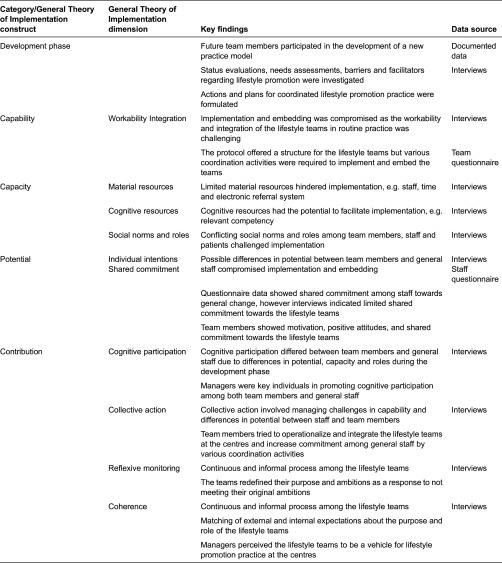
Description of key findings for each category and construct of General Theory of Implementation and their data source

**Table 2.**
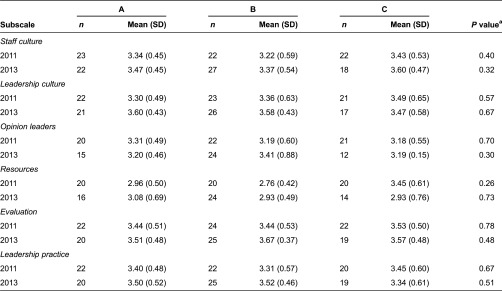
Comparison on organisation readiness to change between primary care centres (A–C)

^a^*P* value for differences between centres.
